# *Bifidobacterium *strains suppress *in vitro *the pro-inflammatory milieu triggered by the large intestinal microbiota of coeliac patients

**DOI:** 10.1186/1476-9255-5-19

**Published:** 2008-11-03

**Authors:** Marcela Medina, Giada De Palma, Carmen Ribes-Koninckx, Miguel Calabuig, Yolanda Sanz

**Affiliations:** 1 Microbial Ecophysiology and Nutrition, Instituto de Agroquímica y Tecnología de Alimentos (CSIC), Apartado 73, 46100 Burjassot, Valencia, Spain; 2 Hospital Universitario La Fe, Avenida Campanar 21, 40009 Valencia, Spain; 3 Hospital General Universitario, Avenida Tres Cruces s/n 46014 Valencia, Spain

## Abstract

**Background:**

Coeliac disease (CD) is an enteropathy characterized by an aberrant immune response to cereal-gluten proteins. Although gluten peptides and microorganisms activate similar pro-inflammatory pathways, the role the intestinal microbiota may play in this disorder is unknown. The purpose of this study was to assess whether the faecal microbiota of coeliac patients could contribute to the pro-inflammatory milieu characteristic of CD and the possible benefits of bifidobacteria.

**Methods:**

The effect of faeces of 26 CD patients with active disease (mean age 5.5 years, range 2.1–12.0 years), 18 symptom-free coeliac disease (SFCD) patients (mean age 5.5 years, range 1.0–12.3 years) on a gluten-free diet for 1–2 years; and 20 healthy children (mean age 5.3 years, range 1.8–10.8 years) on induction of cytokine production and surface antigen expression in peripheral blood mononuclear cells (PBMCs) were determined. The possible regulatory roles of *Bifidobacterium longum *ES1 and *B. bifidum *ES2 co-incubated with faecal samples were also assessed *in vitro*.

**Results:**

Faeces of both active CD and SFCD patients, representing an imbalanced microbiota, significantly increased TNF-α production and CD86 expression in PBMCs, while decreased IL-10 cytokine production and CD4 expression compared with control samples. Active CD-patient samples also induced significantly higher IFN-γ production compared with controls. However, *Bifidobacterium *strains suppressed the pro-inflammatory cytokine pattern induced by the large intestinal content of CD patients and increased IL-10 production. Cytokine effects induced by faecal microbiota seemed to be mediated by the NFκB pathway.

**Conclusion:**

The intestinal microbiota of CD patients could contribute to the Th1 pro-inflammatory milieu characteristic of the disease, while *B. longum *ES1 and *B. bifidum *ES2 could reverse these deleterious effects. These findings hold future perspectives of interest in CD therapy.

## Background

Coeliac disease (CD) is an enteropathy characterized by an aberrant immune response to ingested wheat-gluten proteins (gliadins) and related prolamins of rye and barley, occurring in genetically predisposed (HLA-DQ2/DQ8) individuals. The pathogenesis of CD involves interaction with genetic, immunological and environmental factors. HLA-DQ2/DQ8 molecules of antigen-presenting cells bind and present gluten peptides to lamina propria CD4+ T cells, triggering a T helper 1 (Th1) biased immune response, mainly with interferon gamma (IFN-γ) production, which enhances tumour necrosis factor alpha (TNF-α) production and plays a crucial role in damaging the intestinal mucosa [1,2]. In addition, events leading to CD involve activation of innate immunity mediated by interleukin (IL)-15, and are characterized by expansion of intraepithelial TCRγ/δ + and CD+8 TCRα/β + lymphocytes, which are cytotoxic for epithelial cells and also contribute to tissue damage [3]. The intestinal inflammatory milieu characteristic of CD patients depends on the pro-inflammatory cytokines produced during abnormal response to gluten, involving several intracellular signal transduction pathways, such as nuclear factor *kappa *(NF-κ) B, the interferon regulatory factor (IRF)-1 and signal transducer and activator of transcription [4-6]. NFκB pathway is a crucial target in the propagation of inflammatory responses triggered by cytokines (TNF-α and IFN-γ) and microbial pathogens recognised by Toll-like receptors located in intestinal epithelial and innate immune cells [7]. IκB, a strong regulator of NFκB, is induced by lypopolysaccharide of Gram-negative bacteria, as well as by TNF-α, leading to transcription of genes that contribute to the inflammatory process. Type I interferon IRF-α, which is a cytokine produce by infected cells through the NFκB pathway, induces IFN-γ production and thereby IRF-1 expression, promoting a Th1 response in the CD small intestinal mucosa [4,8]. Increased production of pro-inflammatory cytokines by cells of the innate immune system could also favour the recruitment of lymphocytes into the lamina propria and epithelium, contributing to full expression of the disease [6]. These pathological mechanisms lead to typical CD lesions, characterized by a massive intraepithelial infiltration of lymphocytes, crypt hyperplasia and villous atrophy [1]. Although CD is considered to be the commonest lifelong digestive disorder, the only therapeutic alternative available for CD patients is adherence to a strict gluten-free diet. Poor compliance and associated complications of the disease demand alternative therapeutic strategies.

There is a lack of research into the role of the intestinal microbiota in CD [9] despite the fact gliadin peptides and microorganisms seem to activate similar pro-inflammatory pathways. There have been recent reports of alterations in the composition of the faecal and duodenal microbiota of CD children in comparison with healthy controls [10,11]. *Bifidobacterium *populations were significantly lower in faecal samples of active CD children and also tended to be lower in biopsies when compared with control subjects ([11], Nadal, Medina, Donat, Ribes-Koninckx, Calabuig & Sanz, unpublished). Specific *Bifidobacterium *strains have been acknowledged for their anti-inflammatory and regulatory properties by inducing IL-10 production and regulating the Th1/Th2 balance [12,13]. This has led to certain strains being proposed for use as probiotics, to treat or prevent chronic inflammatory conditions like inflammatory bowel diseases but not CD [9,14].

The aim of the present work was to assess whether alterations in microbiota of the large intestine, corresponding to children with active and non-active CD, could contribute to activate immune responses and induce the pro-inflammatory milieu associated with CD *in vitro *using peripheral blood-mononuclear-cells. In addition, the potential role that selected *Bifidobacterium *strains can play in suppressing the intestinal pro-inflammatory milieu common to these patients was evaluated, as well as their possible mechanism of action.

## Methods

### Subjects and faecal sampling

Altogether 64 children were included in the study: 26 CD patients with active disease (mean age 5.5 years, range 2.1–12.0 years) on a normal gluten-containing diet, 18 symptom-free coeliac disease (SFCD) patients (mean age 5.5 years, range 1.0–12.3 years) on a gluten-free diet for 1–2 years, and 20 healthy children (mean age 5.3 years, range 1.8–10.8 years) without known food intolerance. CD was diagnosed on the basis of clinical symptoms, positive serology markers (antigliadin and antitransglutaminase antibodies) and signs of severe enteropathy by duodenal biopsy examination and positive response to a gluten-free diet. SFCD patients showed negative serology markers and normal duodenal mucosal villous architecture. The children included in the study were not treated with antibiotics for at least one month before the sampling time. The study was conducted in accordance with the ethical rules of the Helsinki Declaration (Hong Kong revision, September 1989), following the EEC Good Clinical Practice guidelines (document 111/3976/88 of July 1990) and current Spanish law which regulates clinical research in humans (Royal Decree 561/1993 regarding clinical trials). Children were enrolled in the study after written informed consent obtained from their parents.

Faecal samples were collected from the three groups of children under study (2 g wet weight), diluted 10-fold in phosphate-buffered saline (PBS, 130 mM sodium chloride, 10 mM sodium phosphate, [pH 7.2]) and homogenized in a Lab Blender 400 stomacher for 3 min (Seward Medical London, UK). Aliquots of this dilution were kept at -80°C for further immunologic studies.

### Bacterial strains and culture conditions

The strains *Bifidobacterium longum *ES1 (CECT 7347) and *Bifidobacterium bifidum *ES2 (CECT 7365) used in the present study were isolated from faeces of healthy babies under breast-milk feeding as described elsewhere [15]. Bifidobacteria were identified at species level by partial sequencing of the 16S rRNA gene amplified with the primers Y1 and 1401R for *B. longum *ES1 and 27F and 1401R for *B. bifidum *ES2 [16,17] and the *tuf *gene amplified with primers BIF-1 and BIF-2 as described elsewhere [18]. Additional primers (27f, Y1, 530f and U-968f) were used for sequencing in an ADN ABI 3700 automated sequencer (Applied Biosystem, Foster City, CA).

Strains were routinely grown in de Man, Rogosa and Sharpe (MRS) broth (Scharlau Chemie S.A., Barcelona, Spain) supplemented with 0.05% (w/v) cysteine (Sigma, St. Louis, MO) (MRS-C) and incubated at 37°C under anaerobic conditions (AnaeroGen; Oxoid, Basingstoke, UK) for 22 h. Cells were harvested by centrifugation (6,000 g for 15 min) during stationary growth phase, washed twice in phosphate buffered saline (PBS, 130 mM sodium chloride, 10 mM sodium phosphate, pH 7.4), and re-suspended in PBS containing 20% glycerol. Aliquots of these suspensions were frozen in liquid nitrogen and stored at -80°C until used. The number of live cells after freezing and thawing was determined by colony-forming unit (CFU) counting on MRS-C agar after 48 h incubation. These constituted the live-cell suspensions used in co-stimulating assays. For all strains tested, more than 90% cells were alive upon thawing and no significant differences were found during storage time (4 months). One fresh aliquot was thawed for every new experiment to avoid variability in the cultures between experiments.

### Isolation and stimulation of peripheral blood mononuclear cells

Peripheral blood mononuclear cells (PBMCs) were isolated from heparinized peripheral blood of four healthy volunteers (median age 30 years, range 24–40 years) as previously described [12]. Briefly, PBMCs were isolated by centrifugation over a Ficoll density gradient (Amersham Biosciences, Piscataway, NJ), and adjusted to 1 × 10^6 ^cells/ml in RPMI 1640 (Cambrex, New York, USA), supplemented with 10% foetal bovine serum (FBS) (Gibco, Barcelona, Spain), 2 mM L-glutamine, 100 *μg*/ml streptomycin and 100 U/ml penicillin (Sigma). PBMCs were incubated in 24-well flat-bottom polystyrene microtitre plates (Corning, Madrid, Spain) and stimulated by either faeces (30 μl), bifidobacterial cell suspensions (10^6 ^CFU/ml) or their combination, at 37°C under 5% CO_2 _for 24 h. Bifidobacterial cell suspensions were washed and re-suspended in fresh PBS prior use for PBMC stimulation. Bacterial growth was not detected during co-incubation of neither faeces or bifidobacterial cell suspensions with PBMCs as determined by colony-forming unit (CFU) counting on Wilkins-Chalgren agar for quantification of total anaerobs (Oxoid, Hampshire, England) and MRS-C agar. Purified lipopolysaccharide (LPS) from *E. coli *O111:B4 (Sigma, St. Louis, MO) was used at a concentration of 1 μg/ml as a positive control. Non-stimulated PBMCs were also evaluated as controls of basal cytokine production and cell-surface marker expression. To investigate the possible involvement of the NK-κB pathway on the immune effects of faeces and bifidobacteria the stimulation of PBMCs was also carried out in the presence of 20 μg/ml lactacystin (Sigma, St. Louis, MO), which is a specific inhibitor of this pathway. All reagents were tested by the E-toxate test for LPS (Sigma) and shown to be below the detection limit (2 pg/ml). Every fraction used as stimulant was assayed in duplicate. Cell-culture supernatants were collected by centrifugation, fractionated in aliquots, and stored at -20°C until cytokines were analysed.

### Cytokine determinations by enzyme-linked immunosorbent assay (ELISA)

Cytokine concentrations of supernatants were measured by ELISA using the Ready SET Go! Kit (BD-Bioscience, San Diego, CA). The pro-inflammatory cytokines TNF-α and INF-γ and the regulatory cytokine IL-10 were analysed. The detection procedures were according to the manufacturer's instructions. The sensitivity of assays for each cytokine was as follows: 4 pg/ml for IFN-γ and TNF-α, and 2 pg/ml for IL-10.

### PBMC surface phenotyping and flow cytometric analyses

To evaluate the effects of the faeces, bifidobacterial suspensions and the combination of both on PBMC surface antigen expression, cells of 1 ml well-culture were removed by scraping and incubated with FITC-labelled anti-human CD4, CD8 and CD86 antibodies for 30 min, according to the manufacturer's instructions (eBioscience, San Diego, CA). Then, cells were washed twice, re-suspended in ice-cold PBS and analyzed by flow cytometry on EPICS^® ^XL-MCL flow cytometer (Beckman Coulter, Florida), setting the 0.22 μl filter that eliminates bacteria. Data were analyzed with the System II V.3 software (Beckman Coulter, Florida). Every sample was assayed in duplicate.

### Statistical analyses

Statistical analyses were carried out with Statgraphics plus 5.1 software (Manugistics, Rockville, MD, USA). Significant differences between means were established by ANOVA with post hoc Fisher's least significant difference (LSD) test at *P *< 0.05. Data are expressed as mean and standard deviation (SD) of duplicate measures determined in four independent experiments.

## Results and discussion

### Cytokine patterns induced by faeces of CD patients on PBMCs

**Figure 1:**
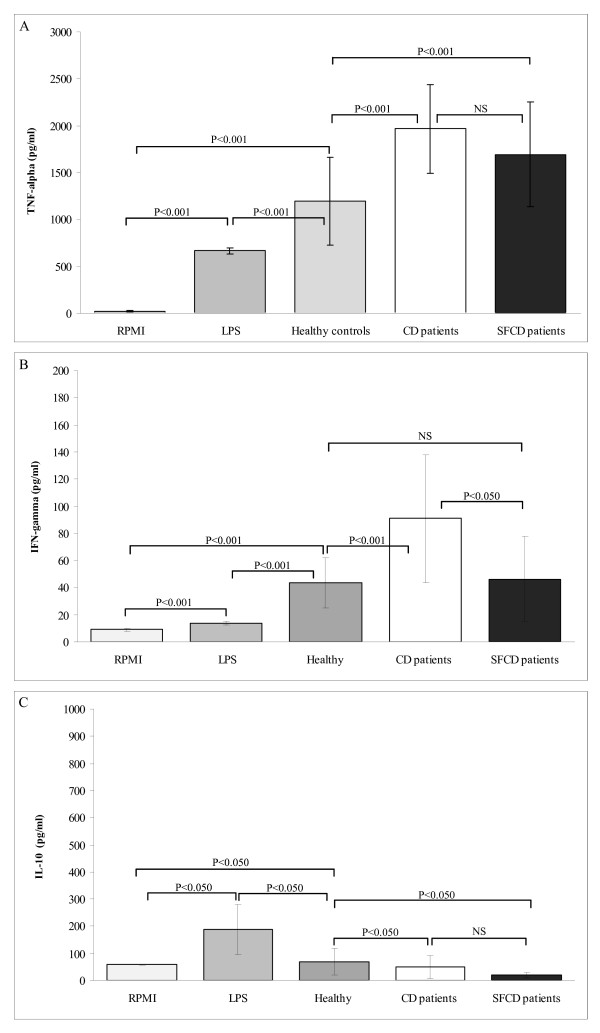
**Cytokine production by peripheral blood mononuclear cells stimulated with faecal samples from active CD patients, symptom free (SF) CD patients and healthy controls.** Panel A, TNF-α production; Panel B, IFN-γ production; Panel C, IL-10 production. Results are expressed as mean ± SD of duplicate measurements determined in four independent experiments. Significant differences between means were established by ANOVA with post hoc Fisher's least significant difference (LSD) test at *P *< 0.05. NS, not significant.

The cytokine production patterns induced by faeces of active CD patients, SFCD patients and age-matched controls in PBMCs are shown in Fig. 1. TNF-α production was significantly higher when cells were stimulated with faeces from both active and SFCD patients as compared to controls (*P *< 0.001; Fig 1A). IFN-γ production was also significantly higher when cells were stimulated with faeces of active CD patients than with those of healthy controls (*P *< 0.001; Fig 1B). By contrast, faeces of healthy controls induced significantly higher IL-10 production than those of active CD patients and, particularly, of SFCD patients (*P *< 0.050; Fig. 1C). Therefore, the immunostimulating effects of faeces of CD patients produced a pro-inflammatory milieu similar to that associated with this disorder, characterized by an increase in IFN-γ and TNF-α production and deficient counter-regulatory mechanisms [2,19]. A Th1 response dominated by high levels of IFN-γ has been reported in the small intestine of untreated CD patients and in the mucosa of treated patients, following culture *in vitro *with gliadin [20] as well as in intraepithelial lymphocytes isolated from untreated coeliac mucosal samples [19,21]. Previously detected differences in the microbiota structure between CD patients and healthy controls could be responsible for the production of the cytokine pattern characteristic of the disease (Nadal et al., unpublished). It has been estimated that bacterial components constitute a major percentage (more than 50%) of faecal solids, representing one of the main bioactive compounds given the high intestinal bacterial numbers reached in the colon (10^11^–10^12 ^CFU per gram of faeces) [22]. In addition, microbial-derived metabolites could contribute indirectly to the detected immunostimulating effects of faeces [23]. In particular, the microbiota of active CD patients was characterized by a significant decrease in the proportions of total Gram-positive bacteria and *Bifidobacterium*, and an increase in *Bacteroides *when compared with SFCD patients and controls. CD patients with active and inactive disease also showed lower ratios of Gram-positive to Gram-negative bacteria when compared with healthy controls (Nadal et al., unpublished). *Bifidobacterium *strains have generally been regarded as anti-inflammatory and beneficial gut microbes, whereas certain species of *Bacteroides *have been shown to trigger inflammation involved in chronic inflammatory bowel diseases [24,25]. In the case of CD, it has been suggested that infections, as well as the overgrowth of opportunistic pathogens, may initiate or contribute to the pathological process by increasing the production of inflammatory mediators. Such mediators include TNF-α and IFN-γ, which are known to increase permeability and could, in turn, favour the access of higher antigen loads (gliadin and microbial) to the submucosa [9]. Furthermore, increased production of pro-inflammatory cytokines like TNF-α and IL-8 through activation of cells of the innate immune system (monocytes, macrophages and dendritic cells) is thought to contribute to the pathogenesis of the disease by promoting lymphocyte recruitment into the lamina propria [6]. IFN-γ could also be involved in T-lymphocyte recruitment and exert an additive effect to that of TNF-α on T-cell migration [26,27]. In addition, IFN-γ has been shown to exert a synergic effect with gliadin peptides, inducing activation of blood monocytes and increasing TNF-*α *production [6]. Therefore, both the imbalanced gut microbiota and gliadins could exert a synergistic effect and stimulate the release of pro-inflammatory cytokines from mucosa innate immune cells, thus contributing to the recruitment of T cells to the submucosa and the full expression of the disease [6,9]. The lower induction of IL-10 production stimulated by faeces of active CD and SFCD patients may also reflect a defect in their ability to counteract the pro-inflammatory responses resulting from alterations in their intestinal microbiota, in addition to those derived from genetic factors [19]. SFCD patients' faeces induce lower IL-10 production, even after following a long-term gluten-free diet. This indicates that these individuals are also more prone to immune dysregulation against a noxious stimulus than age-matched healthy subjects due to changes in the intestinal ecosystem. Increased levels of both IL-10 and IFN-γ have been reported in small intestinal biopsies and intraepithelial lymphocytes isolated from untreated coeliac mucosa [19,21]. Likewise, higher levels of IL-10 mRNA transcripts have been found in untreated coeliac mucosa *in vivo *when compared to treated CD patients and controls [2]. Nevertheless, the ratio between mRNA levels for IL-10 and IFN-γ, as well as that of FoxP3-expressing cells and IFN-γ, was significantly lower in untreated and inflamed CD mucosa than in controls. This would suggest that although these high levels of IL-10 and regulatory T cells reflected a compensatory anti-inflammatory pathway, it was insufficient to suppress the overwhelming Th1 mediated response in active CD [2,19]. In this scenario, immunostimulation triggered by the intestinal microbiota of active and SFCD patients also followed the disease features.

### PBMC surface phenotype induced by faeces of CD patients

**Figure 2:**
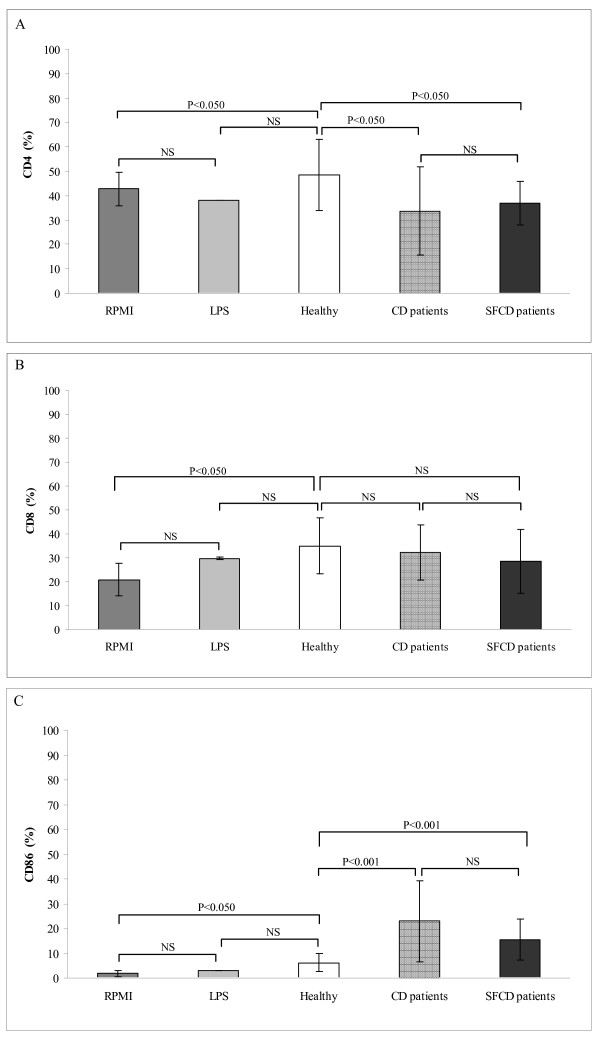
**Expression of surface markers CD4 (Panel A), CD8 (Panel B) and CD86 (Panel C) induced by peripheral blood mononuclear cells stimulated with faecal samples from active CD patients,  symptom-free (SF) CD patients and healthy controls.** Results are expressed as mean ± SD of duplicate measurements determined in four independent experiments. Significant differences between means were established by ANOVA with post hoc Fisher's least significant difference (LSD) test at *P *< 0.05. NS, not significant.

Expression of certain surface molecules in PBMCs indicates their maturation level and may predict the quality of interaction between antigen-presenting cells and T cells and thereby their activation. Fig 2 shows the results of the analysis of PBMCs surface antigen expression induced by faeces from active CD children, SFCD patients and age-matched controls. CD4 expression was significantly lower when PBMCs were stimulated with faeces from active CD patients than with healthy control samples (*P *< 0.050; Fig 2A). CD4 expression was also significantly down-regulated when PBMCs were stimulated with samples from SFCD patients as compared to healthy controls (*P *< 0.050, Fig 2A). In contrast, CD8 expression did not differ significantly under the effects of faecal samples from the three groups of children under study (Fig 2B). These results suggest that expression of the co-receptor molecule CD4 could also be under the stimulating effects of the intestinal microbiota in controls and CD patients. In controls, higher CD4 expression could lead to an increase in the CD4+ regulatory T cell subpopulation involved in IL-10 production, which is important in maintaining tolerance to enteric bacteria and dietary antigens [25]. This is in agreement with the higher production of IL-10 by PBMCs stimulated with faecal samples from healthy individuals compared to CD-patient stimulated samples.

In contrast, expression of the co-stimulatory molecule CD86 increased significantly when PBMCs were stimulated with faeces from both active CD and SFCD patients (*P *< 0.001) compared with healthy controls (Fig 2C). CD86 expression levels differed after stimulation with the three faecal-sample types, with the highest to lowest levels corresponding to active CD faecal samples, followed by SFCD-patient samples, and healthy control samples, respectively. Evidently, the intestinal microbiota of both types of CD patients triggered a higher expression of the costimulatory molecule CD86, which plays a major role in initiating immune responses. CD86, together with CD80 expression on antigen-presenting cells, are essential for T-cell activation through antigen-specific stimulation, contributing to Th1 response [28]. Stimulation of monocyte-derived dendritic cell (DC) maturation by microbial strains or derived products has also been shown to induce expression of CD86, CD83 and CD40 by both commensal and pathogenic bacteria [29,30]. However, molecules involved in activation of Th1 cells were predominantly expressed on DC exposed to pathogens, parallel to higher pro-inflammatory cytokine production such as TNF-α [29]. In general, Gram-negative bacteria have been shown much more effective in up-regulating maturation markers of DCs than lactic acid bacteria at lower concentrations [31]. Gliadin is also known to induce phenotypic and functional maturation of monocytes, as well as monocyte-derived DCs [6]. Up-regulation of surface expression of CD80, CD83, CD86 and CD40 was induced by stimulation of blood mononcytes with gliadin peptides, particularly in combination with IFN-γ [6]. Therefore, this is the first reported evidence that gut microbiota stimulus, together with gliadin, could contribute to monocyte maturation, thereby, influencing T-cell interaction and activation.

### Bifidobacteria suppress the pro-inflammatory cytokine pattern induced in PBMCs by faeces of CD patients

**Figure 3:**
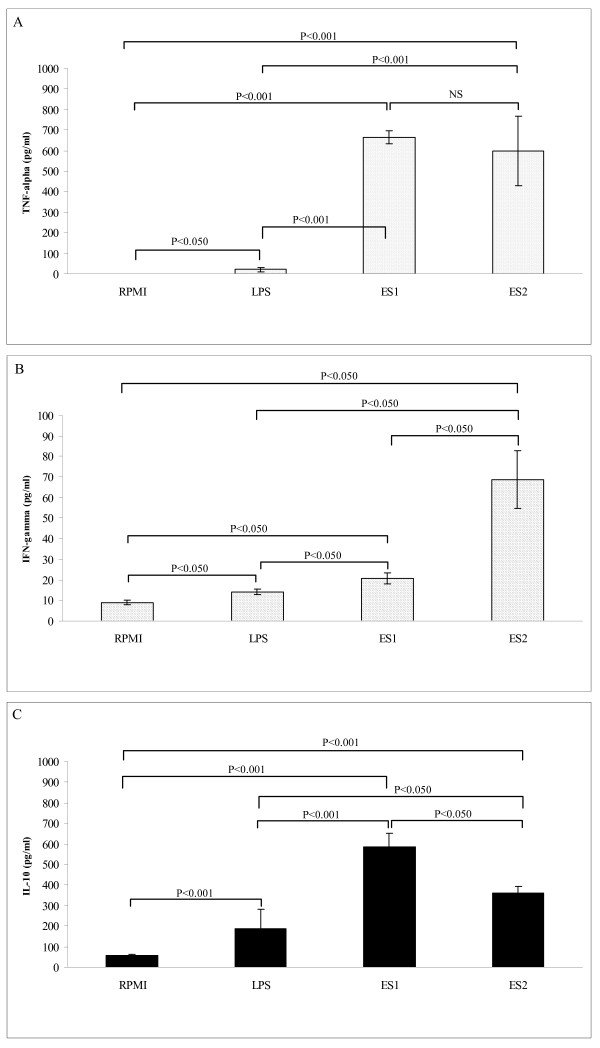
**Cytokine production by peripheral blood mononuclear cells stimulated with live bacteria of *Bifidobacterium longum *ES1 and *B. bifidum *ES2.** Panel A, TNF-α production; Panel B, IFN-γ production; Panel C, IL-10 production. Results are expressed as mean ± SD of duplicate measures determined in four independent experiments. Significant differences between means were established by ANOVA with post hoc Fisher's least significant difference (LSD) test at *P *< 0.05. NS, not significant.

The *Bifidobacterium *strains included in this study were selected on the basis of their ability to induce pro- and anti-inflammatory cytokine production by PBMCs (Fig. 3). Both strains *B. longum *ES1 and *B. bifidum *ES2 stimulated the production of significantly higher levels of TNF-α and IL-10 (P < 0.001; Fig 3A) than non-stimulated cells. Both strains displayed a similar ability to induce TNF-α production, as reported in previous comparative studies [12]. In contrast, *B. longum *ES1 induced significantly higher levels (*P *< 0.050; Fig 3C) of IL-10 production than *B. bifidum *ES2 but lower levels (P < 0.050; Fig 3B) of IFN-γ production. Thus, *B. longum *ES1 has greater potential to counteract a Th1-biased response by inducing high production of the regulatory cytokine IL10 and low production of the Th1 cytokine IFN-γ. *Bifidobacterium *strains are generally regarded as less pro-inflammatory than *Lactobacillus*, more often inducing lower Th1-type cytokine production and a T regulatory phenotype based on induction of high IL-10 production [13,25]. Furthermore, a recent comparative study of the different immunomodulatory properties of bifidobacteria has shown that this trait is strain-dependent, thus different strains can divert immune response either towards a Th1 pro-inflammatory or a regulatory profile, highlighting the importance of careful selection for probiotic applications [12]. The *Bifidobacterium *strains included in this study also tended to reduce PBMC surface antigen expression markers, including CD4, CD8 and CD86, when used as stimuli, although effects were not statistically significant (data not shown). According to our results, none of the probiotic strains of the VSL#3 product modified CD8 expression in dendritic cells (DCs) [25]. In contrast, there are previous reports of *Bifidobacterium *species-specific effects on expression of DC surface markers, demonstrating general increases in CD86 and CD83 expression [32].

**Figure 4:**
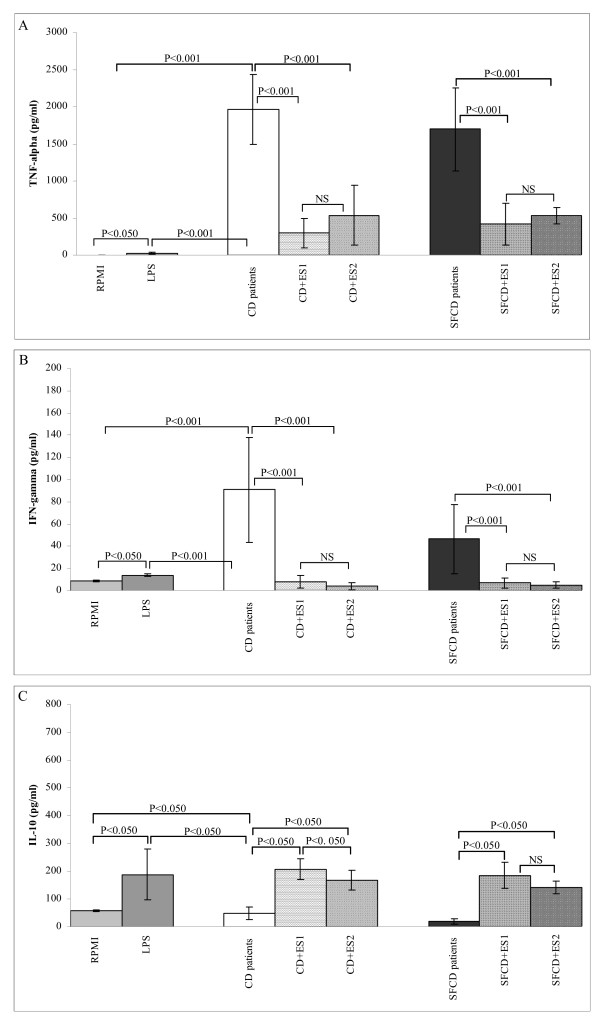
**Cytokine production by peripheral blood mononuclear cells co-stimulated with faecal samples from either active CD patients or symptom free (SF) CD patients together with live bacteria of either *Bifidobacterium longum *ES1 and *B. bifidum *ES2.** Panel A, TNF-α production; Panel B, IFN-γ production; Panel C, IL-10 production. Results are expressed as mean ± SD of duplicate measurements determined in four independent experiments. Significant differences between means were established by ANOVA with post hoc Fisher's least significant difference (LSD) test at *P *< 0.05. NS, not significant.

The ability of *B. longum *ES1 and *B. bifidum *ES2 strains to revert the pro-inflammatory cytokine profile induced by faeces of CD children was also evaluated for potential probiotic applications (Fig. 4). When *B. longum *ES1 was used as stimulus together with the faeces of active CD and SFCD patients, TNF-α (*P *< 0.001) and IFN-γ (*P *< 0.001) production was significantly lower than cytokine levels produced under exclusive stimulation with both CD patient faeces (Fig. 4A and 4B). Similar results were obtained with *B. bifidum *ES2 (Fig. 4A and 4B). Thus, both *Bifidobacterium *stains could counteract the production of both pro-inflammatory cytokines (TNF-α and IFN-γ) triggered by the altered microbiota, and thus contribute to normalization of the intestinal inflammatory milieu. Although *Bifidobacterium *cell suspensions were able to induce TNF-α when used as unique stimuli, they could counter-regulate TNF-α production when added together with patient's faeces probably as a result of their interactions or synergic effects with other components present in faecal samples.

Regulatory IL-10 cytokine production increased significantly (*P *< 0.050; Fig 4C) by co-stimulation with *B. longum *ES1 and *B. bifidum *ES2 together with faeces of both patients (Fig. 4C). *B. longum *ES1 increased IL-10 production to a significantly higher extent than *B. bifidum *ES2 (*P *< 0.050) according to the data obtained using pure cultures of these strains. This would suggest a more powerful regulatory role for the former strain. IL-10 plays an important role in regulating the inflammatory cascade in the intestinal mucosa by its action on antigen-presenting cells via inhibition of cytokine synthesis. IL-10 inhibits the production of Th1 pro-inflammatory cytokines and particularly IFN-γ and in turn TNF-α, which is induced by IFN-γ. Mice genetically deficient in IL-10 develop chronic enterocolitis caused by an unregulated Th1 response to endogenous bacterial flora, which could be counteracted by a strain of *Lactococcus lactis *secreting recombinant IL-10 [33]. IL-10 administration is also reported to exert beneficial therapeutic effects in Crohn's disease patients by intravenous administration [34]. In the context of CD, recombinant human IL-10 has been shown to suppress Th1-mediated immune responses to gliadin in both treated and untreated coeliac mucosa via down regulation of antigen presentation, reduction of T-cell infiltration and activation, and inducing a long-lasting hyporesponsiveness in gliadin-specific T cells [2]. However, the clinical usefulness of IL-10 is limited for technical reasons related to organ-specific delivery and, therefore, a therapeutic approach based on probiotic strains triggering IL-10 production would overcome these limitations and provide new therapeutic perspectives [35].

*B. longum *ES1 and *B. bifidum *ES2 co-incubated with the faeces of CD patients led to slightly lower expression of surface markers on PBMCs, particularly in the case of CD4 and CD86. The down-regulatory effects of *B. bifidum *ES2 seemed to be stronger than those of *B. longum *ES1 but none of these differences were statistically significant (data not shown).

### Cytokine production but not PBMC maturation depends on NFkB pathway

**Figure 5:**
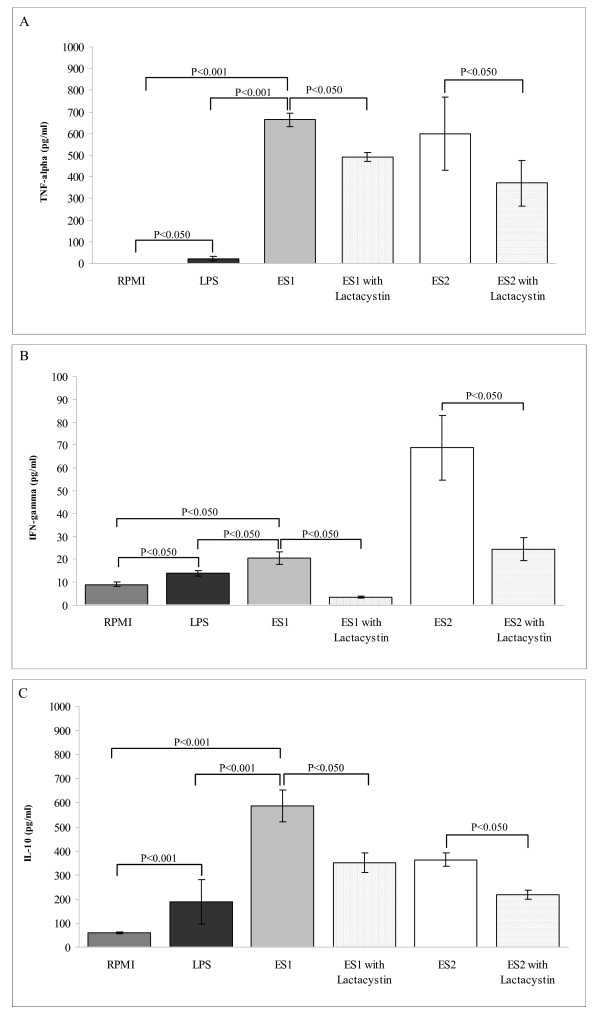
**Cytokine production by peripheral blood mononuclear cells stimulated with live bacteria of *Bifidobacterium longum *ES1 and *B. bifidum *ES2 in the presence or absence of lactacystin.** Panel A, TNF-α production; Panel B, IFN-γ production; Panel C, IL-10 production. Results are expressed as mean ± SD of duplicate measures determined in four independent experiments. Significant differences between means were established by ANOVA with post hoc Fisher's least significant difference (LSD) test at *P *< 0.05. NS, not significant.

Cytokine production (TNF-α, IFN-γ, and IL-10) by PBMCs stimulated with faeces of the three groups of children under study was completely abolished in the presence of lactacystin, an inhibitor of the NFκB pathway (data not shown). TNF-α, IFN-γ and IL-10 production by PBMCs stimulated by pure cultures of *Bifidobacterium *strains was also inhibited on average 33.0% (SD 9.9), 75.0% (SD 18.3) and 50.5% (SD 14.9), respectively, in the presence of lactacystin (Fig 5). These results indicate that NFκB pathway is involved in the immune effect of the total intestinal microbiota, as well as of the tested *Bifidobacterium *strains. Interaction of the gut microbiota with innate immune cells through pattern recognition receptors, like Toll-like receptors (TLRs), is considered to be the starting point of immunity, sensing the environment and informing the immunocompetent cells to respond properly to pathogens or harmless antigens [7]. Common to all TLR is the activation of NF-κB transcription, leading to upregulation of major histocompatibility complexes and costimulatory proteins, as well as production of pro-inflammatory cytokines and chemokines (TNF-*α*, IL-1β, and IL-8) and recruitment of other immune cells. Specific TLRs including TLR4, which recognizes LPS from Gram-negative bacteria, also activate interferon regulatory factor 3 (IRF3) or IRF7, leading to the production of type I IFNs, such as IFNα, that stimulate IFN-γ synthesis [36]. TLR4 and TLR2 mRNA and proteins are up-regulated in the duodenal mucosa of CD patients with active and non-active disease compared with controls [37], which could contribute to amplifying the immune response derived from stimulation by altered intestinal microbiota in these patients. The NFkB signal transduction pathway is also involved in gliadin-induced cytokine production by monocytes from CD patients [6] as well as in increased intestinal permeability and zonulin production triggered by gliadins in the intestinal epithelium [38].

CD4, CD8 and CD86 expression was slightly reduced by stimulation with every faecal sample and *Bifidobacterium *strains in the presence of lactacystin, but the differences were not significant (data not shown). This would suggest that a different activation mechanism, and not the NFκB pathway, mediates surface antigen expression in the assayed conditions. By contrast, supernatants of a *Bifidobacterium breve *strain are known to influence maturation of monocyte-derived dendritic cells by means of NFkB pathway but not survival and IL-10 production [30].

In summary, this is the first report that the content of the large intestine of both active and SFCD patients, representing imbalanced gut microbiota, increases pro-inflammatory cytokine production and CD86 activation marker expression in PBMCs as compared to healthy controls. Likewise it decreases anti-inflammatory IL-10 cytokine production, which reflects the Th1 pro-inflammatory milieu characteristic of CD. Moreover, *Bifidobacterium *strains with immunoregulatory properties have been shown to suppress the pro-inflammatory cytokine pattern induced by the altered colonic microbiota of CD patients and strengthen the immune defences of active and SFCD patients against noxious antigens from the intestinal lumen. This mechanism of action could complement the *in vitro *protective effects exerted by other *Bifidobacterium *strain against permeability changes induced by gliadin peptides [39].

## Conclusion

The intestinal microbiota of CD patients could contribute to the Th1 pro-inflammatory milieu characteristic of the disease, while strains of *B. longum *and *B. bifidum *could reverse these deleterious effects. Thus, the results reported here offer novel perspectives in the therapy of CD based on immune modulation by the use of specific probiotic strains.

## Abbreviations

CD: coeliac disease; DC: dendritic cells; IFN-γ: interferon gamma; IL: interleukin; NF-κ: nuclear factor kappa; PBMCs: peripheral blood mononuclear cells; SFCD patient: symptom-free CD patient; TNF-α: tumour necrosis factor alpha.

## Competing interests

There are non-financial competing interests (political, personal, religious, ideological, academic, intellectual, commercial or any other) to declare in relation to this manuscript

## Authors' contributions

GDP and MM equally contribute to data acquisition and analysis. MC and CRK contribute to patient recruitment, clinic assessments and sampling; and YS contributed to conception and design of the study, wrote the manuscript and gave final approval. All authors have read and approved the final version of the manuscript.
